# MRI free water mediates the association between water exchange rate across the blood brain barrier and executive function among older adults

**DOI:** 10.1162/imag_a_00183

**Published:** 2024-06-05

**Authors:** Colleen Pappas, Christopher E. Bauer, Valentinos Zachariou, Pauline Maillard, Arvind Caprihan, Xingfeng Shao, Danny J.J. Wang, Brian T. Gold

**Affiliations:** Department of Neuroscience, College of Medicine, University of Kentucky, Lexington, KY, United States; Department of Behavioral Science, College of Medicine, University of Kentucky, Lexington, KY, United States; Department of Neurology, University of California at Davis, Davis, CA, United States; Center for Neurosciences, University of California at Davis, Davis, CA, United States; The Mind Research Network, Albuquerque, NM, United States; Laboratory of FMRI Technology (LOFT), Stevens Neuroimaging and Informatics Institute, Keck School of Medicine, University of Southern California, Los Angeles, CA, United States; Department of Radiology, College of Medicine, University of Kentucky, Lexington, KY, United States; Sanders Brown Center on Aging, University of Kentucky, Lexington, KY, United States; Magnetic Resonance Imaging and Spectroscopy Center, University of Kentucky, Lexington, KY, United States

**Keywords:** aging, blood brain barrier, diffusion prepared arterial spin labeling, diffusion weighted imaging, free water, executive function

## Abstract

Vascular risk factors contribute to cognitive aging, with one such risk factor being dysfunction of the blood brain barrier (BBB). Studies using non-invasive magnetic resonance imaging (MRI) techniques, such as diffusion prepared arterial spin labeling (DP-ASL), can estimate BBB function by measuring water exchange rate (kw). DP-ASL kw has been associated with cognition, but the directionality and strength of the relationship is still under investigation. An additional variable that measures water in extracellular space and impacts cognition, MRI free water (FW), may help explain prior findings. A total of 94 older adults without dementia (Mean age = 74.17 years, 59.6% female) underwent MRI (DP-ASL, diffusion weighted imaging (DWI)) and cognitive assessment. Mean kw was computed across the whole brain (WB), and mean white matter FW was computed across all white matter. The relationship between kw and three cognitive domains (executive function, processing speed, memory) was tested using multiple linear regression. FW was tested as a mediator of the kw-cognitive relationship using the PROCESS macro. A positive association was found between WB kw and executive function [F(4,85) = 7.81,*p*< .001, R^2^= 0.269; β = .245,*p*= .014]. Further, this effect was qualified by subsequent results showing that FW was a mediator of the WB kw-executive function relationship (indirect effect results: standardized effect = .060, bootstrap confidence interval = .0006 to .1411). Results suggest that lower water exchange rate (kw) may contribute to greater total white matter (WM) FW which, in turn, may disrupt executive function. Taken together, proper fluid clearance at the BBB contributes to higher-order cognitive abilities.

## 1 Introduction

The study of vascular contributors to cognitive impairment and dementia (VCID) has gained increased attention (Corriveau et al., 2016;Zlokovic et al., 2020) as poorer vascular health (e.g., small vessel disease, hypertension) can contribute to cognitive deficits (Hamilton et al., 2021;Ou et al., 2020). VCID includes impaired blood brain barrier (BBB) function, which has been tied to cognitive performance as well as cognitive impairment (Montagne et al., 2015;Verheggen et al., 2020;Zachariou et al., 2023). The BBB is a non-fenestrated structure composed of astrocytes, pericytes, and endothelial cells which protects the brain from harmful substances (Keaney & Campbell, 2015). Furthermore, the BBB is integral to glymphatic system function, a key component in waste clearance from the brain (Iliff et al., 2012;Keaney & Campbell, 2015). Physiological changes in the BBB with age can include increased pericyte damage (Cicognola et al., 2023), increased endothelial cell senescence (Kiss et al., 2020), and altered aquaporin-4 expression in astrocytes (Zeppenfeld et al., 2017), all of which could have a negative effect on cognition.

Advancements in magnetic resonance imaging (MRI) techniques have led to improvements in the*in vivo*measurement of BBB health. Currently, the gold standard for examining BBB permeability is gadolinium-based dynamic contrast-enhanced (DCE) MRI (Chagnot et al., 2021;Varatharaj et al., 2019). DCE-MRI with gadolinium has been used to evaluate permeability among older adults with and without cognitive impairment (Montagne et al., 2015;Verheggen et al., 2020). An age-associated increase in white matter (WM) and grey matter BBB permeability has been found for cognitively unimpaired older adults (Verheggen et al., 2020) while greater BBB permeability in structures such as the hippocampus has been associated with cognitive impairment (Montagne et al., 2015;Nation et al., 2019).

Although DCE-MRI based studies have been important in understanding the functionality of the BBB in aging and disease states, utilizing non-contrast MRI methods can be advantageous for this growing body of research. One such method is diffusion-prepared arterial spin labeling (DP-ASL) MRI which has been developed to estimate the regional water exchange rate (kw) across the BBB (Shao et al., 2019). DP-ASL assesses kw by using multiple diffusion weightings to estimate differences in magnetically-tagged water (ASL perfusion) signal between the capillary and parenchymal compartments. The rate of water exchange between these compartments is derived using a two-compartment model of the ASL signal with single-pass approximation (SPA;St Lawrence et al., 2012). When directly comparing DP-ASL to DCE-MRI techniques, positive correlations between the two measures were observed in only 3 of 10 regions of interest (Shao et al., 2020). This could indicate that different physiological features of the BBB are being captured by DP-ASL than DCE-MRI, such as potentially glymphatic system-based clearance versus large molecule infiltration.

Thus, as a potentially sensitive, non-invasive metric of BBB function, it is important to gain a greater understanding of the interplay between BBB kw and cognition in older adults. A relationship between BBB kw and cognitive performance has been reported (Gold et al., 2021;Shao et al., 2019;Uchida et al., 2022;Zachariou et al., 2023). Specifically, BBB kw has been associated with memory and executive function performance (Gold et al., 2021;Uchida et al., 2022;Zachariou et al., 2023). In these studies, higher kw (a greater water exchange rate) and better memory or executive function was observed, although results were region specific and revealed small effects in some cases. The opposite relationship (i.e., higher kw and poorer cognitive performance), however, has been found among individuals at risk for cerebral small vessel disease (Shao et al., 2019). Further, another study measuring water permeability via water extraction with phase contrast arterial spin tagging MRI revealed an association between greater water permeability and poorer memory and global cognition (Lin et al., 2021).

In contrast to the moderate strength of relationships observed between BBB kw and cognition, and some ambiguity about the direction of those relationships, another MRI measure of water diffusion, namely diffusion MRI-based free water (FW), has been more strongly correlated with cognition (Gustavson et al., 2023;Maillard et al., 2019;Maillard, Hillmer, et al., 2022). FW has more consistently shown a negative direction of association with cognitive function (Gullett et al., 2020;Maillard et al., 2019). Specifically, high FW has been linked to poorer performance on tasks that test speeded response, executive function, or reasoning abilities (Berger et al., 2022;Gullett et al., 2020;Gustavson et al., 2023) as well as poorer performance on memory and global cognitive function tasks (Maillard et al., 2019).

Given that both MRI-based FW and kw relate to cerebral water diffusion mechanisms, and that greater FW accumulation has been consistently linked to poorer cognitive outcomes, it is plausible that FW is a mediator of the kw-cognition relationships. Here, we tested this hypothesis in a sample of community-dwelling, older adults without dementia. Relationships between kw and three cognitive measures (executive function, processing speed, and memory) were first tested. The association between FW across all WM and kw across the whole brain (WB) was also examined. We then investigated FW as a potential mediator between whole brain kw and executive function, processing speed, or memory. Our analyses focused on whole brain measures of kw and FW to understand global relationships between these two relatively new metrics with each other and with cognition.

## 2 Materials and Method

### 2.1 Participants

All study participants were recruited from the Sanders Brown Center on Aging (SBCoA) Longitudinal cohort (Schmitt et al., 2012). Participants were enrolled in one of two neuroimaging studies being conducted at the SBCoA: the University of Kentucky Alzheimer’s Disease Research Center (UK-ADRC) Biomarker Core study or the Standardized Centralized Alzheimer’s Related Neuroimaging (SCAN) Initiative. Inclusion criteria for enrollment into the SBCoA Longitudinal cohort are a minimum of 60 years of age, cognitive and neurological normality at the enrollment examination (based on clinical consensus diagnosis performed by the UK-ADRC Clinical Core); designated informant for structured interviews; and willingness to undergo annual cognitive testing and physical and neurological examinations. Individuals with a history of head injury, major psychiatric illness or current substance abuse, medical illnesses that are nonstable, impairing, or that have an effect on the CNS, chronic infectious diseases, stroke or transient ischemic attack, encephalitis, meningitis, or epilepsy are excluded from participation in this cohort.

Additional exclusion criteria for the present study were diseases affecting the blood (e.g., anemia, kidney disease, heart disease), MRI-related contraindications (i.e., pacemakers, metal fragments, metal implants, claustrophobia) or significant brain abnormalities discovered upon imaging. Additional inclusion criteria for the present study were the absence of dementia at the time of the most recent MRI scan and the availability of an MRI diffusion-prepared pseudo continuous arterial spin labeling (DP-ASL) scan and a diffusion weighted scan. Ninety-six participants met initial eligibility criteria for this study. Two participants were excluded based on their MRI data: one due to hydrocephalus not known at the time of enrollment and another for being an outlier in the measure of average white matter FW. This resulted in a total sample size of 94 participants for our study. All participants provided informed consent under a protocol approved by the Institutional Review Board of the University of Kentucky.

### 2.2 MRI acquisition

Participants were scanned at the University of Kentucky’s Magnetic Resonance Imaging and Spectroscopy Center (MRISC) using a 3 Tesla Siemens Magnetom Prisma MRI scanner with a 64-channel head coil. Data from the following sequences were used for the current study: (1) a 3D, T1-weighted magnetization prepared rapid gradient echo (T1) sequence; (2) a 3D gradient-and-spin-echo (GRASE) diffusion-prepared pseudo-continuous arterial spin labeling sequence; (3) a spin-echo, echo-planar multi-shell diffusion sequence; and (4) a spin-echo, echo-planar diffusion weighted sequence with reverse phase-encoding direction from the main (3) diffusion sequence. Data from several other sequences were collected during the scanning session, but pertained to unrelated scientific questions and are not further outlined here.

A 3D T1-weighted, magnetization-prepared rapid gradient echo (MPRAGE) sequence covering the whole brain was acquired using a generalized autocalibrating partial parallel acquisition acceleration factor (factor 2) in the sagittal plane with 1 mm^3^spatial resolution. Other parameters varied slightly based upon whether participants were enrolled in the UK-ADRC Biomarker Core Longitudinal MRI battery [Multiecho MPRAGE (MEMPRAGE): Number of echoes: 4, 256 x 256 x 176 mm acquisition matrix (176 slices), repetition time (TR) = 2,530 ms, first echo time (TE1) = 1.69 ms, echo time spacing echo spacing (ΔTE = 1.86 ms), flip angle = 7°, scan duration = 5.88 min (n = 52)] or enrolled in the SCAN Initiative [MPRAGE: 256 x 240 x 208 mm acquisition matrix (208 slices), TR = 2,300 ms, (TE = 2.98 ms) flip angle = 9°, scan duration = 5.20 min (n = 42)].

The following parameters were used for DP-ASL sequence collection: Field of view = 224 mm, matrix size = 64 × 64, 12 slices (10% oversampling), resolution = 3.5 × 3.5 × 8 mm, TR = 4 s, TE = 36.5 ms, label/control duration = 1,500 ms, centric ordering, optimized timing of background suppression for gray matter (GM) and white matter (WM), and scan duration = 10 min (Shao et al., 2018). Arterial transit time (ATT) and kw were measured following a two‐stage approach. First, ATT was acquired with 15 repetitions during the flow encoding arterial spin tagging (FEAST) scan at post‐labeling delay (PLD) = 900 ms and diffusion weighting (b‐value) of 0 and 14 s/mm^2^(Wang et al., 2007). Next, the kw metric was calculated from scans acquired at PLD = 1,800 ms and diffusion weighting b = 0 and 50 s/mm^2^to measure the labeled blood arriving at the microvascular compartment. Twenty repetitions were acquired for each b‐value of the kw scan. CBF was quantified from control-label difference images at the 1,800 ms PLD as previously described (Shao et al., 2019).

The main MRI diffusion scan was acquired using 126 different directions [2 mm isotropic voxels, 232 x 232 x 162 mm acquisition matrix (81 slices), TE = 71 ms, TR = 3,400 ms, posterior-to-anterior phase encoding direction, multislice acceleration = factor 3, phase partial Fourier = 6/8, and scan duration = 7.45 min], divided among 4 b-values [0 s/mm^2^(12 directions), 500 s/mm^2^(6 directions), 1,000 s/mm^2^(48 directions), and 2,000 s/mm^2^(60 directions)]. A 28-s reversephase-encoding scan (anterior-to-posterior phase encoding direction) was acquired [2 mm isotropic voxels, 232 x 232 x 162 mm acquisition matrix (81 slices), TE = 71 ms, TR = 3,400 ms, multislice acceleration = factor 3, phase partial Fourier = 6/8, and 2 b-values (0 and 2,000 s/mm^2^)] immediately following the main diffusion scan to correct for susceptibility-induced distortions in the main diffusion scan. Only the non-diffusion weighted (b = 0) images were used for this purpose as recommended by FSL’s topup (Andersson et al., 2003).

### 2.3 MRI processing

#### 2.3.1 T1 weighted image

The T1-weighted MPRAGE was processed in FreeSurfer (version 6.0) using the recon–all option (Dale et al., 1999;Desikan et al., 2006). The skull-stripped T1-weighted image (whole brain) was used for registration with the DP-ASL sequence. The T1-weighted image was also used to estimate total intracranial volume and lateral ventricle size.

#### 2.3.2 Diffusion-prepared pseudo-continuous arterial spin labeling

The DP-ASL data were processed with the Water Exchange Quantification Toolbox (version 1) using principles described byShao et al. (2019). To summarize, control/label images were subtracted to obtain perfusion images following motion correction with SPM12 (The Wellcome Centre for Human Neuroimaging). Temporal fluctuations were minimized using principal component analysis (Shao et al., 2017). Maps were created for kw, ATT, and CBF. The tissue and capillary compartments of the ASL signal were separated by a small diffusion gradient of 50 s/mm^2^for the sequence. The kw map was calculated by a total‐generalized‐variation (TGV) (Spann et al., 2020) regularized SPA model (St Lawrence et al., 2012) using the tissue (or capillary) fraction of the ASL signal at the PLD of 1,800 ms, incorporating ATT, T1 of arterial blood and brain tissue as inputs for the algorithm (Shao et al., 2019). Arterial blood T1 was calculated as 1.66 s based on prior CBF research (Lu et al., 2004).

Next, the T1-weighted image was registered to the DP-ASL M0 as described previously (Zachariou et al., 2023). Due to the low resolution of the DP-ASL images, a series of steps were required for registration. In brief, the T1-weighted image was first aligned with the center of the DP-ASL M0 using the AFNI program @Align_Centers. The center-aligned T1-weighted image brain was then registered to the DP-ASL M0 by align_epi_anat.py using an edge-based approach which optimized alignment between the two images. Then, the resulting output matrix was used to align the original images to one another. The T1-weighted image was visually inspected for accuracy of registration with the M0 image. In the case that registration was poor or failed, the nudge feature in FSLeyes was utilized and applied to the alignment matrix. Once optimal registration was achieved, the whole brain T1-weighted image mask was rescaled to the resolution of the DP-ASL M0 image. Overlap between the T1-weighted image mask and the kw map was calculated. As the DP-ASL sequence did not capture the entire brain, a threshold of 80% coverage was utilized to ensure optimal coverage. The mean whole brain (WB) kw (min^-1^) was used to measure the rate of water diffusion across the BBB in subsequent multiple linear regression (Section 2.5.1) and mediation (Section 2.5.2) analyses.

**Fig. 1.**
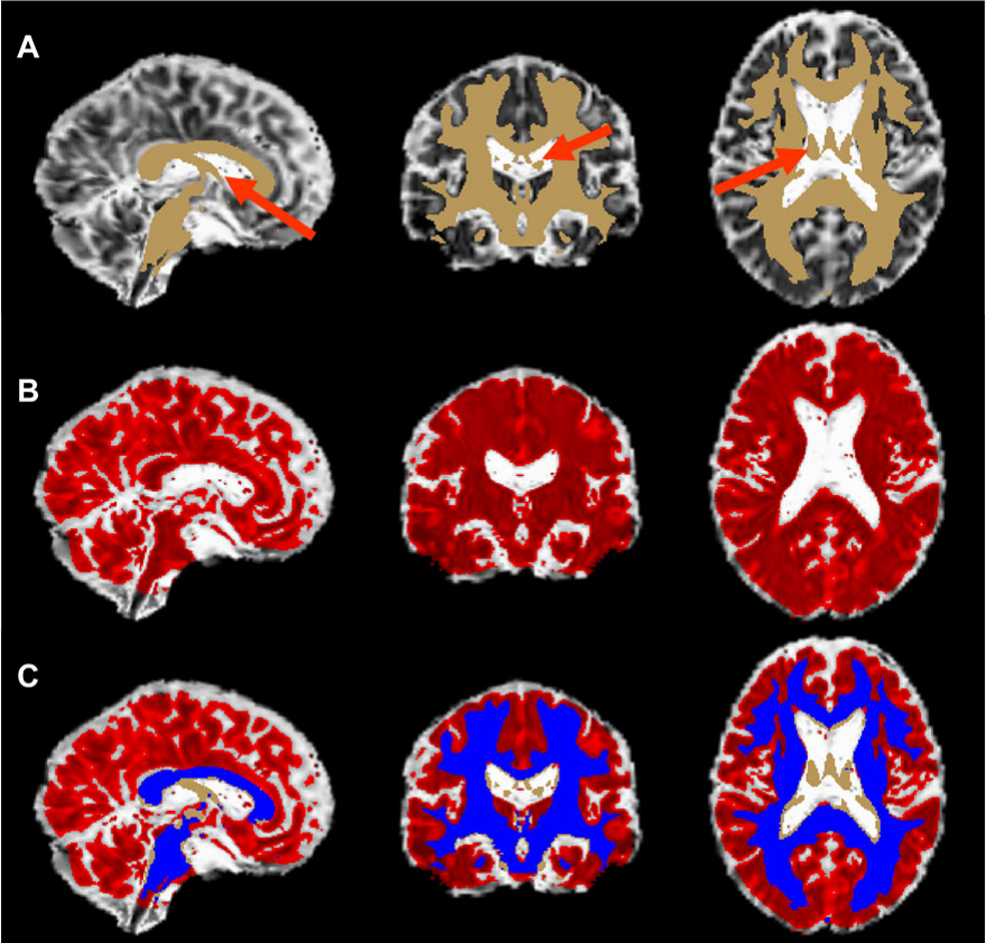
A visual representation of the methodology used to obtain the white matter free water mask. The template mask for white matter free water is displayed in Panel (A) (tan) overlaid on the free water map in MNI space. Red arrows in Panel (A) show voxels that are CSF, or small fibers intermingled with CSF, for a representative participant. The free water fraction map for the entire brain that has been thresholded to free water fraction values above the 80th percentile (Panel (B); red) is overlaid on the non-thresholded free water map in MNI space. Panel (C) depicts the overlap between the white matter mask and the 80% thresholded free water fraction map (blue). The voxels in the blue mask represent the whole-brain white matter free water used in analyses. Areas in Panel (C) where there is no overlap (tan) are excluded in the mean free water calculation.

A similar approach was taken to measure the mean kw in regions of interest (ROIs). FreeSurfer-derived masks were created for cortical and subcortical regions (using recon-all), and lobar masks were created as outlined in FreeSurfer documentation (https://surfer.nmr.mgh.harvard.edu/fswiki/CorticalParcellation). The masks were then registered to the DP-ASL M0 image using the whole brain alignment matrix (Section 2.3.2; paragraph 2). Following registration, grey matter masks were eroded by one voxel to account for partial volume effects. Then, all masks were rescaled to the resolution of the DP-ASL M0 image. We selected five ROIs based on prior work (Gold et al., 2021): Frontal lobe, parietal lobe, hippocampus, left precuneus, and right precuneus. To ensure the ROIs selected were adequately captured in the kw maps, a more stringent threshold of 90% overlap between the given ROI mask and the kw map was used. Additionally, to standardize ROI values between participants, kw maps were z-scored relative to each participant’s average whole brain kw output. The mean kw (z-scored) in the selected ROIs was used in linear regression analyses (Section 2.5.1). A representative kw map from one participant can be found inSupplementary Figure 1.

#### 2.3.3 Diffusion weighted imaging free water

The preprocessing pipeline for diffusion MRI data in this study has been described in detail in our previous work (Bauer et al., 2021,^2022^). Briefly, each participant’s main diffusion data was corrected for susceptibility-induced field distortions with FSL’s topup (Andersson et al., 2003) using the participant’s reverse phase-encoding diffusion scan. All diffusion data were then skull-stripped using BET (Smith, 2002) and non-linearly corrected for participant motion and eddy currents with eddy (Andersson & Sotiropoulos, 2016). Eddy QC tools (Bastiani et al., 2019) were used to assess average head motion across volumes for each participant (average voxel displacement across all voxels within a brain mask relative to the first volume). A threshold of 2 mm was used for exclusion; no participants exceeded this threshold. All diffusion MRI data were also visually examined to ensure quality. Each participant’s motion-corrected diffusion data was used as an input to produce fractional anisotropy (FA) and FW fraction maps.

FA maps were calculated using FSL’s DTIFIT (https://fsl.fmrib.ox.ac.uk/fsl/fslwiki/FDT/UserGuide#DTIFIT). FW fraction maps were calculated using a modified kit developed from the multi-site MarkVCID consortium (https://markvcid.partners.org/;Maillard, Hillmer, et al., 2022;Maillard, Lu, et al., 2022). First, a two-compartment model from the multishell Free Water Diffusion Tensor algorithm (Bauer et al., 2022;Henriques et al., 2017) available through the Diffusion Imaging in Python (DIPY) open-source software library (Garyfallidis et al., 2014) was used to calculate the fractional volume of FW per voxel in the motion-corrected diffusion data, resulting in FW fraction maps. Each participant’s FA map was then aligned (fsl_reg;http://ftp.nmr.mgh.harvard.edu/pub/dist/freesurfer/tutorial_packages/OSX/fsl_501/bin/fsl_reg) to a standard FSL FA template space (FMRIB 1-mm FA template) using linear and nonlinear transformations (Maillard, Lu, et al., 2022). These transformations were then applied to each participants FW fraction map (Maillard, Lu, et al., 2022).

FW fraction values above the 80% threshold were set to zero in the FW fraction map to reduce potential partial volume contamination from cerebral spinal fluid (CSF) as done in our previous work (Bauer et al., 2022). A whole brain white matter mask throughout the brain was defined by thresholding the FSL FA template by a value of 0.3 to further eliminate overlap with CSF (Maillard, Lu, et al., 2022). The mean FW fraction value throughout this whole brain white matter mask (in all non-zero voxels) was then extracted using fslstats (https://open.win.ox.ac.uk/pages/fslcourse/practicals/intro3/index.html) for each participant (Maillard, Lu, et al., 2022). A representative white matter mask and FW fraction map are displayed inFigure 1to illustrate the methodology used. Mean FW fraction values were subsequently used in multiple linear regression (Section 2.5.1) and mediation (Section 2.5.2) analyses.

### 2.4 Neuropsychological testing

All participants underwent neuropsychological testing at the UK ADRC using the National Alzheimer’s Coordinating Center’s Uniform Data Set, version 3 (UDS-3;Besser et al., 2018). Additional UK-ADRC site-specific neuropsychological measures were also collected. For the purposes of the current study, three cognitive domains were tested: executive function, processing speed, and memory. Scores from the Montreal Cognitive Assessment (MoCA;Nasreddine et al., 2005) are also reported as a measure of global cognitive status.

An executive function composite was created by factor score analysis methods (Staffaroni et al., 2021) and included the following neuropsychological tests: Category fluency (animals, vegetables), phonemic fluency (letter F, letter L), digit span backward, and trail making test A and B. The number of correct responses for category fluency, phonemic fluency, and digit span backward were used while the correct number of lines per minute were calculated for the trail making tests. Processing speed was measured by the digit symbol substitution test from the Weschler’s Adult Intelligence Scale Revised (WAIS-R;Wechsler, 1987). The total number of correct responses in a 90-s time frame was scored from the digit symbol substitution test. Memory was assessed by the Craft Story immediate and delayed recall (paraphrase score) and the Benson complex figure delayed recall (total score). Items were z-scored for the sample and then averaged to create the memory composite following the work ofDodge et al. (2020). For all three cognitive domains, higher scores indicate better performance.

### 2.5 Statistical analyses

Statistical analyses were completed in SPSS version 28 (IBM Corp., Armonk, NY, USA), and two-tailed alphas were set at .05. Outliers greater than 3.29 SD from the mean were removed prior to analyses. Multicollinearity was assessed by the variance inflation factor, with a value of less than 5 considered acceptable (Stine, 1995).

#### 2.5.1 Multiple linear regression

Separate multiple linear regression models were used to test the relationship between WB kw (independent variable) and executive function composite, processing speed, and memory composite (dependent variables). Covariates were age (years), sex, and education (years). Similarly, WB CBF and ATT (independent variables) were separately examined as predictors of cognitive performance. We also tested the relationship between five separate ROIs and cognition (executive function, processing speed, and memory) using multiple linear regression analyses with the aforementioned covariates. A Holm-Bonferroni correction (Holm, 1979) for multiple comparison was applied for the cognitive tests. Using this method, the alpha level is adjusted successively by the number of remaining comparisons (i.e., .017, .025, .05 for the three tests in our study).

Multiple linear regression was also used to examine the relationship between WB kw and FW, with age (years) and sex as covariates. To gain a better understanding of the WB kw-FW relationship, we conducted additional follow-up testing with lateral ventricle size used as a covariate to account for neurodegeneration/brain atrophy. Lateral ventricle size was selected as ventricular expansion is a common feature of brain atrophy and degeneration (Apostolova et al., 2012), which is more likely to occur among aging populations. Further, lateral ventricle size was used in place of whole brain volume as it has been correlated with measures of white matter microstructure (Coutu et al., 2016). Lateral ventricle volume was regressed onto the estimated total intracranial volume with the residualized values used for analyses. We selected the residual approach as it better accounts for sex-related differences in brain volume (Voevodskaya et al., 2014).

#### 2.5.2 Mediation

To better understand factors that contribute to the water diffusion-cognition relationship, mediation analyses were conducted using the PROCESS macro (version 4.0) for SPSS (Hayes, 2022). We tested average white matter (WM) FW as a mediator between WB kw and cognitive outcomes (PROCESS Model 4). WB kw and average WM FW were specifically selected for the mediation analyses to obtain further insight about potential global inter-relationships between these measures. Covariates included age, sex, and education and 5,000 bootstrap samples were utilized in all mediation analyses. Follow-up analyses were also conducted using the residualized lateral ventricle volume as an additional covariate.

## 3 Results

Participant characteristics are presented inTable 1. The sample was 74.17 years old on average, and the majority was female (59.6%). Participants were highly educated (16.9 years) and had an average MoCA score of 26.6.

**Table 1.** Participant characteristics (n = 94)

Characteristic	Mean	SD	No (%)
Age	74.17	5.69	
Sex (female)			56 (59.6)
Education ^†^	16.90	2.59	
MoCA ^‡^	26.62	3.00	
Whole brain kw	114.67	24.90	
White matter FW	0.24	0.02	

Whole brain kw is measured in min^-1^.

Note: FW = free water. MoCA = Montreal Cognitive Assessment.

† n = 90;^‡^n = 91.

### 3.1 kw, CBF, ATT, and cognition

Of the 94 participants with kw and free water (FW) data, a subset had cognitive data (Mean days from MRI visit = 60.9, SD = 65.7). Four participants did not have data for the covariate education. Additional missing data were observed for memory (n = 2) and processing speed (n = 1). One outlier (>3.29 SD from the mean) for processing speed was removed from subsequent analyses. This resulted in the following sample size for each cognitive domain: executive function (n = 90), processing speed (n = 88), and memory (n = 88).

**Fig. 2.**
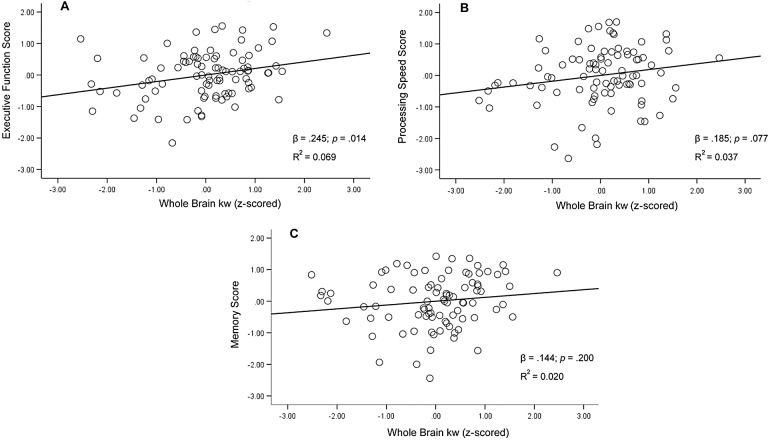
Regression plots for whole-brain kw and executive function composite (A), processing speed (B), and memory composite (C). Covariates in all models included age, sex, and education. For ease of interpretation, figures reflect z-scored values.

Whole brain (WB) kw was tested as a predictor of executive function, processing speed, and memory. WB kw was significantly associated with executive function after controlling for age, sex, and education [F(4,85) = 7.81,*p*< .001, R^2^= 0.269; β = .245,*p*= .014]. As WB kw values increased, better performance on tasks of executive function was observed (Fig. 2A). Results remained significant following Holm-Bonferroni correction. There was a trend toward a WB kw and processing speed relationship after controlling for covariates [F(4,83) = 5.44,*p*< .001, R^2^= 0.208; β = .185,*p*= .077;Fig. 2B]. Similar to executive function, higher kw was associated with better processing speed performance. Conversely, WB kw was not significantly associated with the memory composite after controlling for age, sex, and education [F(4,83) = 1.58,*p*= .187, R^2^= 0.071; β = .144,*p*= .200;Fig. 2C].

To gain a better understanding of how additional metrics of perfusion influence cognition, we tested WB CBF or ATT as predictors of executive function, processing speed, and memory. CBF was associated with executive function [F(4,85) = 7.20,*p*< .001, R^2^= 0.253; β = .230,*p*= .041] and processing speed [F(4,83) = 6.31,*p*< .001, R^2^= 0.233; β = .280,*p*= .016] after controlling for age, sex, and education. However, only processing speed survived multiple comparison correction. Conversely, CBF was not associated with memory [F(4,83) = 1.52,*p*= .203, R^2^= 0.068; β = .151,*p*= .232].

ATT was not associated with executive function [F(4,85) = 5.95,*p*< .001, R^2^= 0.219; β = .065,*p*= .519] after controlling for covariates. Similarly, there was not a relationship present with processing speed [F(4,85) = 4.47,*p*= .003, R^2^= 0.177; β = .010,*p*= .926] or memory [F(4,83) = 1.60,*p*= .182, R^2^= 0.072; β = -.146,*p*= .191].

Relationships between kw in the selected ROIs (frontal lobe, parietal lobe, hippocampus, left precuneus, and right precuneus) and cognitive abilities (executive function, processing speed, and memory) were also tested. A significant relationship was found for parietal lobar kw and memory after controlling for age, sex, and education [F(4,83) = 2.73,*p*= .034, R^2^= 0.116; β = .257,*p*= .016]. As kw in the parietal lobe increased, better memory performance was observed. No other results were significant following Holm-Bonferroni correction. Complete results are reported inTable 2.

**Table 2.** Kw region of interest and cognition results

	Executive function			Processing speed			Memory		
	n	β	*p* -value	n	β	*p* -value	n	β	*p* -value
Region of Interest									
Frontal Lobe	90	.038	.709	88	.069	.660	88	.073	.519
Parietal Lobe	90	.066	.502	88	.022	.829	**88**	**.257**	**.016**
Hippocampus	83	-.044	.668	81	-.172	.127	81	-.169	.130
Left Precuneus	90	.109	.259	88	.081	.420	88	.178	.095
Right Precuneus	90	.067	.493	88	.087	.386	88	.069	.524

Note: Standardized β values are reported in the table. Covariates were age, sex, and education. Results that remained significant following Holm-Bonferroni correction are denoted in bold text.

### 3.2 Average white matter free water and cognition

Total white matter (WM) FW was also tested as a predictor of executive function, processing speed, and memory. After controlling for age, sex, and education, average WM FW was significantly associated with executive function [F(4,85) = 7.93,*p*< .001, R^2^= 0.272; β = -.268,*p*= .012]. Lower WM FW was related to better performance on tasks of executive function. Results remained significant following Holm-Bonferroni correction. WM FW was also associated with processing speed after controlling for covariates [F(4,83) = 6.06,*p*< .001, R^2^= 0.226; β = -.248,*p*= .025], but did not survive multiple comparison correction. A relationship was not observed between WM FW and the memory composite [F(4,83) = 1.61,*p*= .181, R^2^= 0.072; β = -.157,*p*= .188].

### 3.3 kw and average white matter free water

**Fig. 3.**
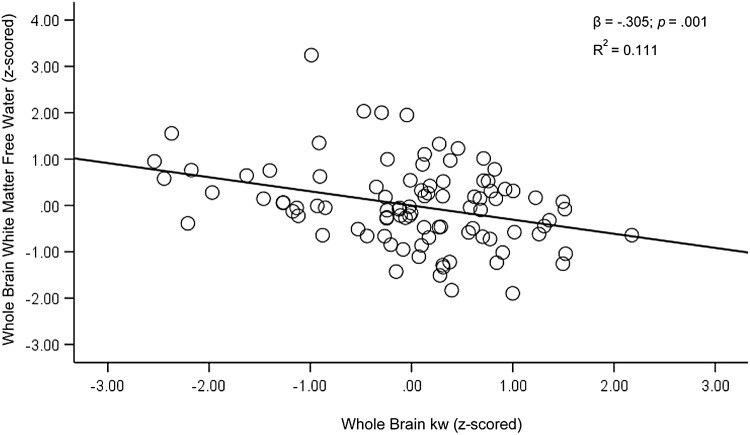
Regression plots for whole brain kw and whole brain white matter free water. Covariates included age, sex, and residualized lateral ventricle size. For ease of interpretation, z-scored values were used.

Next, we tested WB kw as a predictor of FW in total white matter (WM). WB kw was significantly associated with average WM FW after controlling for age and sex [F(3,90) = 10.17,*p*< .001, R^2^= 0.253; β = -.300,*p*= .002]. As kw values increased, FW values decreased (Fig. 3). We also tested the relationship between total WM FW and WB kw with the residualized lateral ventricle size added as a covariate to account for atrophy. Results followed a similar pattern, with higher kw being associated with lower FW [F(4,89) = 9.95,*p*< .001, R^2^= 0.309; β = -.305,*p*= .001].

### 3.4 Mediation

Based on our kw, FW, and cognitive function results, mediation models were implemented. The first model (Model 1) tested the relationship between kw and executive function with FW as the mediator. An indirect effect (standardized effect = .060, bootstrap confidence interval [CI] = .0006 to .1411) was observed from the data, which indicated that FW mediated the relationship between kw and executive function (Supplementary Figure 2&Supplementary Table 1). A direct effect was not observed (standardized β = .186,*p*= .071). Next, we tested the reverse model where kw acted as the mediator between FW and executive function (Model 2). Neither an indirect effect (standardized effect = -.060, bootstrap CI = -.144 to .014) nor a direct effect (standardized β = -.208,*p*= .057) was present.

**Fig. 4.**
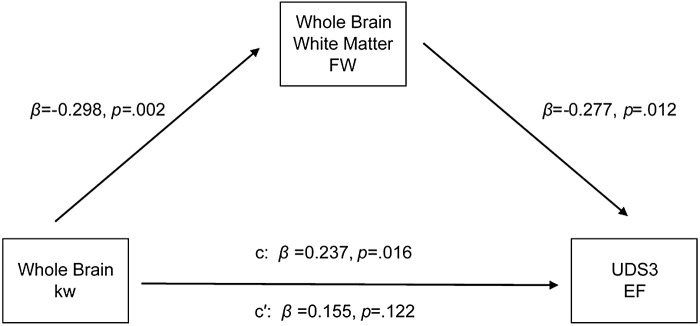
The association between whole-brain kw and executive function composite score, with whole-brain white matter free water as a mediator. An indirect effect was observed for the models, but not a direct effect. Covariates included age, sex, education, and residualized lateral ventricle size.

When adding the residualized lateral ventricle volume as a covariate to Model 1, results remained similar. An indirect effect (standardized effect = .083, bootstrap CI = .0161 to .1639) was observed, showing FW mediates the kw-executive function relationship above and beyond brain atrophy (Fig. 4;Supplementary Table 2). A direct effect was not observed (standardized β = .155,*p*= .122). Testing the reverse model (Model 2) yielded null findings for the indirect effect (standardized effect = -.055, bootstrap CI = .0161 to .1639) but not the direct effect (standardized β = -.277,*p*= .012). Taken together, our results suggest that FW mediates the relationship between kw and executive function such that greater kw may lead to less total WM free water which, in turn, could lead to better executive performance.

Mediation models were also tested with processing speed as the outcome variable. Neither an indirect effect (standardized effect = .059, bootstrap confidence interval [CI] = -.002 to .141) nor direct effect (standardized β = .126,*p*= .240) was observed (Supplementary Table 3). For the reverse model (Model 2) where kw acted as the mediator between FW and processing speed, an indirect effect (standardized effect = -.040, bootstrap CI = -.104 to .016) and a direct effect (standardized β = -.208,*p*= .070) were not present. However, when the residualized lateral ventricle volume was added to the model, an indirect effect (standardized effect = .075, bootstrap CI = .008 to .158) was found, but not a direct effect (standardized β = .105,*p*= .329;Supplementary Table 4). Testing the reverse model (Model 2) yielded null findings for the indirect effect (standardized effect = -.037, bootstrap CI = -.108 to .022), but the direct effect was statistically significant (standardized β = -.255,*p*= .031).

Finally, mediation was tested with memory as the cognitive variable of interest. No significant effects were found for the kw-FW-memory relationship (*p*s > .05). Due to the lack of findings with the multiple linear regression and mediation Model 1, Model 2 and analyses with residualized lateral ventricle volume as a covariate were not tested.

## 4 Discussion

Our study examined the relationship between MRI-based water exchange across the blood brain barrier (BBB) and cognitive performance and whether that relationship is mediated by MRI-based free water (FW). We found a positive relationship between whole brain (WB) kw and executive function. However, the kw-executive function relationship was mediated by FW across all white matter (WM), where higher FW was associated with poorer executive function performance. The moderate effects of kw on cognition reported in the literature may therefore be in part explained by the complex interplay between dysfunctional BBB kw and the accumulation of free water in extracellular space.

Previous work has reported moderate relationships between kw and cognition, sometimes with different direction of relationship, depending upon the regions studied (Gold et al., 2021;Shao et al., 2019;Uchida et al., 2022;Zachariou et al., 2023). In the current study, we found that higher WB kw was related to better executive function performance, but not processing speed or memory. We also found that WB CBF was related to processing speed, whereas WB ATT was not associated with any of our cognitive measures. From these results, executive function appeared to be more impacted by kw than other metrics of cerebral perfusion (i.e., CBF, ATT). Our ROI results indicated a selective, positive relationship between kw in the parietal lobe and performance on the MEM composite. The discrepant findings between studies and across brain regions could be due in part to differences in cognitive status of the samples being studied and cognitive domains explored. However, another potential contributing factor to the discrepant findings may be that a previously unexplored third variable (such as MRI FW) acts as a mediator of the relationship between BBB kw and cognition.

Our results indicate that FW mediates the relationship between WB kw and executive function. The directionality of the relationship suggests that lower WB kw predicts higher WM FW, with higher WM FW then predicting poorer executive function. Although our results are novel, they build upon those from a previous study, which found that FW mediated the relationship between white matter hyperintensity volume and several measures of cognitive function (Maillard et al., 2019). In the present study, our mediation-based results held when controlling for lateral ventricle size, which was used as a proxy for macrostructural brain atrophy (which would be primarily driven by neurodegeneration). This shows that our results are not purely due to ventricular expansion, but rather may more closely reflect extracellular free water dynamics within white matter.

It should also be noted that in our study FW was measured in WM whereas whole brain kw was measured in both grey and white matter. This is in part due to signal-to-noise ratio constraints with DP-ASL imaging in WM. While this does not provide an exact one-to-one comparison between neuroimaging modalities, it is feasible that water exchange rate across the BBB near neuronal cell bodies, glia, and neuropil may be associated with extracellular fluid levels around axons. Whether these physiological processes are correlated between cell bodies and their specific connected or neighboring axons is an important topic for future research.

There are several biological mechanisms that could explain the association between BBB kw and FW, many of which involve the glymphatic system. The glymphatic system functions to maintain interstitial fluid balance and clear waste products from the brain (Hablitz & Nedergaard, 2021;Iliff et al., 2012). Waste clearance includes, but is not limited to, molecules implicated in Alzheimer’s disease, particularly extracellular proteins, such as amyloid beta (Aβ) (Iliff et al., 2012;Nauen & Troncoso, 2022). We recently found that higher BBB kw in multiple brain regions was associated with higher CSF concentration of Aβ-42 (less Aβ cerebral deposition), suggesting that high kw may in part be indexing proper glymphatic clearance functions (Gold et al., 2021).

Accordingly, one potential explanation of our current findings is that low kw could in part reflect toxic accumulation of Aβ, which then contributes to degradation of WM, resulting in higher extracellular FW and poorer cognitive performance. This possibility is consistent with results showing that Aβ oligomers can directly damage oligodendrocytes (Horiuchi et al., 2012;Xu et al., 2001), prevent proper myelination (Horiuchi et al., 2012), and impair lipid function (Kaya et al., 2020). In human research, evidence of a link between Aβ and WM structure comes from work showing that low levels of CSF Aβ-42 (high cerebral Aβ binding) are associated with low diffusion MRI fractional anisotropy (Gold et al., 2014). Further, in positron emission tomography (PET)/MRI studies, Aβ-positive individuals showed higher mean diffusivity (Caballero et al., 2020) and lower fractional anisotropy over time in multiple major white matter tracts (Vipin et al., 2019).

Alternatively, or in addition to, the deleterious effects of Aβ on WM, dysregulation of BBB astrocytic water channels could result in FW accumulation, contributing to poorer cognitive performance. Aquaporin 4 (AQP4) channels are abundant in astrocytes and allow for water transport across the BBB (Nagelhus & Ottersen, 2013;Salman et al., 2022). Dysregulation of AQP4 may therefore result in a diminished capacity for efficient BBB-related water exchange. For example, AQP4 knockout mice have been found to have a poorer BBB water exchange rate (Zhang et al., 2019) and greater brain interstitial fluid content (Gomolka et al., 2023) than their wildtype counterparts.

In the case of Aβ accumulation and/or AQP4 dysfunction, the resulting interstitial fluid accumulation from WM injury would be expected to contribute to poorer cognitive function. Indeed, higher levels of FW in the extracellular space are known to be associated with poorer cognitive performance (Dumont et al., 2019;Kamagata et al., 2022;Maillard et al., 2019). This relationship likely reflects that FW metric indexes neurodegeneration, neuroinflammation, and breakdown of cellular membranes (Altendahl et al., 2020;Nakaya et al., 2022), all of which would be expected to negatively impact cognitive performance.

In our study, we found executive function to be most affected by the FW mediation. However, a similar relationship was not found with memory. In general, it appears that WM damage tends to be more disruptive of speeded tasks used in both processing speed and executive function domains compared to non-speeded tasks used to assess other cognitive domains such as episodic memory (Borghesani et al., 2013;Gustavson et al., 2023;Vernooij et al., 2009). Consistent with these results, a recent study found that FW contributed to fluid (i.e., executive function, working memory, processing speed) but not crystallized (i.e., verbal abilities) measures of cognition (Gullett et al., 2020).

There are several strengths of the current study. This is the first study to our knowledge to examine the complex interplay between DP-ASL kw and FW metrics and their relation to cognitive function in aging. As such, a global approach to mediation analyses was adopted, focusing on whole brain measures of kw and total FW in WM to gain a better understanding of overall contributions of water clearance dynamics to cognitive abilities. To ensure that our findings were not simply driven by neurodegeneration effects captured by FW, we completed follow-up analyses that controlled for atrophy. CSF partial volume effects for FW were also stringently controlled for in order to accurately capture extracellular free water in WM only.

Limitations of this study include the cross-sectional design, which does not allow for causal inference. In addition, our sample consists of primarily White, highly educated participants which may limit generalizability of our findings. Future studies should attempt to replicate our analyses in more diverse cohorts. While novel MRI measures were used to estimate water exchange across the BBB and extracellular free water concentration, these specific physiological functions remain only heuristics of their corresponding MRI metrics at this point. Additional animal model work is needed to specify the precise physiological processes associated with kw and FW and how they interact with each other. Further development of these novel MRI measures will also help overcome technological limitations that could limit the biological specificity of these biomarkers. Nevertheless, our work represents a starting point for exploring the complex interplay between glymphatic clearance and free water accumulation via human*in vivo*MRI studies.

In conclusion, our results suggest that FW appears to be an important mediator in the relationship between BBB water exchange (kw) and cognitive function. In cases where water is not properly moved across the BBB, FW accumulates, which can damage WM and contribute to poorer executive function. Future research should examine the kw-FW-cognitive relationship longitudinally, with an emphasis on biological factors that further explain extracellular water accumulation and clearance.

## Supplementary Material

Supplementary Material

## Data Availability

All data and code will be made available without reservation following a formal data-sharing agreement with the corresponding author. Data requests for summary measure results available for UK-SBCoA Longitudinal cohort can be made by completing a Data Request Form found at:https://medicine.uky.edu/centers/sbcoa/data-sample-request. Data requests for summary measures will be reviewed by the UK-ADRC Executive Committee.
