# Supplementary material for: MRI free water mediates the association between water exchange rate across the blood brain barrier and executive function among older adults
Source: Imaging Neurosci (Camb). 2024 Jun 5;2:imag-2-00183. doi: 10.1162/imag_a_00183 (PMC11211995; doi:10.1162/imag_a_00183)
Supplement: Supplementary Material [file imag_a_00183-supp.pdf]

Supplementary Table 1. Mediation Results for Executive Function

	Unstandardized β	95% CI		Standardized β	p-value
		LL	UL		
**Model 1: Mediator FW**					
kw → FW (Path a)	-.0002	-.0003	-.0001	-.287	.004
FW → EF (Path b)	-11.47	-23.31	.369	-.208	.057
kw → EF (Path c)	.009	.002	.015	.245	.014
Indirect Effect (Path ab)[a]	.002	.000	.005	.060	-
Direct Effect (Path c′)	.006	-.001	.014	.186	.071
**Model 2: Mediator kw**					
FW → kw (Path a)	-516.35	-863.57	-169.13	-.325	.004
kw → EF (Path b)	.006	-.001	.014	.186	.071
FW → EF (Path c)	-14.80	-26.23	-3.37	-.268	.012
Indirect Effect (Path ab)[a]	-3.33	-7.94	.727	-.060	
Direct Effect (Path c′)	-11.47	-23.31	.369	-.208	.057

Note. Mediation was tested in separate models with executive function as the outcome variable. Covariates in all models include age, sex, and education.

[a]Bootstrapped confidence intervals (5,000) are reported for the indirect effect results.

CI = confidence interval; LL = lower limit; UL = upper limit; FW = free water; EF = executive function.

Supplementary Table 2. Mediation Results for Executive Function with Lateral Ventricle Size

	Unstandardized β	95% CI		Standardized β	p-value
		LL	UL		
**Model 1: Mediator FW**					
kw → FW (Path a)	-.0002	-.0003	-.0001	-.298	.002
FW → EF (Path b)	-15.33	-27.22	-3.43	-.277	.012
kw → EF (Path c)	.008	.002	.015	.237	.016
Indirect Effect (Path ab)[a]	.003	.0006	.006	.083	-
Direct Effect (Path c′)	.005	-.002	.012	.155	.122
**Model 2: Mediator kw**					
FW → kw (Path a)	-564.00	-920.36	-207.64	-.355	.002
kw → EF (Path b)	.005	-.002	.012	.155	.122
FW → EF (Path c)	-15.62	-26.05	-5.18	-.313	.004
Indirect Effect (Path ab)[a]	-3.04	-7.88	1.28	-.055	
Direct Effect (Path c′)	-15.33	-27.22	-3.43	-.277	.012

Note. Mediation was tested in separate models with executive function as the outcome variable. Covariates in all models include age, sex, education, and lateral ventricle size (residualized values).

[a]Bootstrapped confidence intervals (5,000) are reported for the indirect effect results.

CI = confidence interval; LL = lower limit; UL = upper limit; FW = free water; EF = executive function.

Supplementary Table 3. Mediation Results for Processing Speed

	Unstandardized β	95% CI		Standardized β	p-value
		LL	UL		
**Model 1: Mediator FW**					
kw → FW (Path a)	-.0002	-.0003	-.0001	-.283	.005
FW → Processing Speed (Path b)	-140.62	-293.05	11.82	-.208	.062
kw → Processing Speed (Path c)	.079	-.009	.167	.185	.077
Indirect Effect (Path ab)[a]	.025	-.001	.062	.059	-
Direct Effect (Path c′)	.054	-.037	.145	.126	.240
**Model 2: Mediator kw**					
FW → kw (Path a)	-502.84	-852.27	-153.40	-.318	.005
kw → Processing Speed (Path b)	.054	-.037	.145	.126	.240
FW → Processing Speed (Path c)	-167.78	-313.53	-22.03	-.248	.025
Indirect Effect (Path ab)[a]	-27.16	-68.30	11.43	-.040	
Direct Effect (Path c′)	-140.62	-293.05	11.82	-.208	.070

Note. Mediation tested in separate models with processing speed as the outcome variable. Covariates in all models include age, sex, and education.

[a]Bootstrapped confidence intervals (5,000) are reported for the indirect effect results.

CI = confidence interval; LL = lower limit; UL = upper limit; FW = free water.

Supplementary Table 4. Mediation Results for Processing Speed with Lateral Ventricle Size

	Unstandardized β	95% CI		Standardized β	p-value
		LL	UL		
**Model 1: Mediator FW**					
kw → FW (Path a)	-.0002	-.0003	-.0001	-.294	.003
FW → Processing Speed (Path b)	-172.97	-329.77	-16.17	-.255	.031
kw → Processing Speed (Path c)	.077	-.011	.165	.180	.085
Indirect Effect (Path ab)[a]	.032	.003	.070	.069	
Direct Effect (Path c′)	.045	-.046	.136	.105	.284
**Model 2: Mediator kw**					
FW → kw (Path a)	-553.57	-912.60	-194.55	-.350	.003
kw → Processing Speed (Path b)	.045	-.046	.136	.105	.329
FW → Processing Speed (Path c)	-197.82	-346.27	-49.36	-.292	.010
Indirect Effect (Path ab)[a]	-24.84	-71.03	15.62	-.040	
Direct Effect (Path c′)	-172.97	-329.77	-16.17	-.255	.030

Note. Mediation was tested in separate models with processing speed as the outcome variable. Covariates in all models include age, sex, education, and lateral ventricle size (residualized values).

[a]Bootstrapped confidence intervals (5,000) are reported for the indirect effect results.

CI = confidence interval; LL = lower limit; UL = upper limit; FW = free water.

Supplementary Figure 1. kw Map

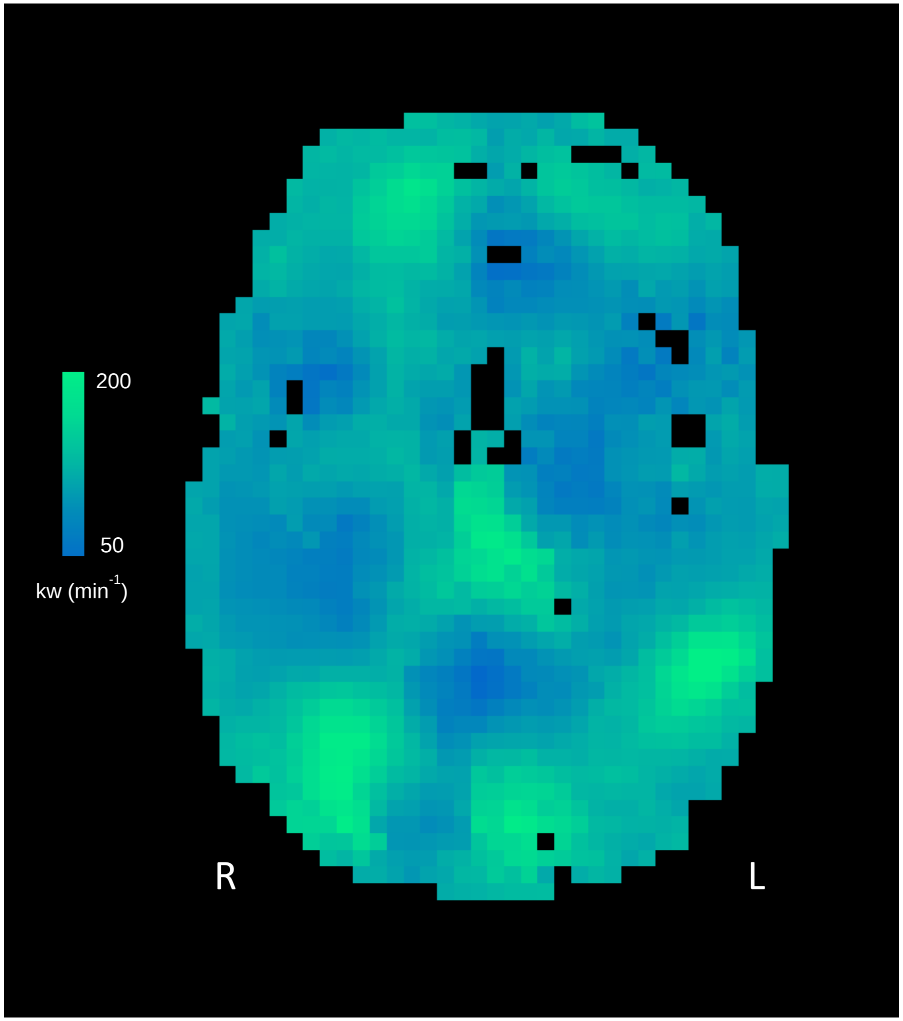

Note. A representative kw map for a participant**.**
R = right. L = left.

Supplementary Figure 2. Mediation Model for Water Diffusion and Executive Function without Lateral Ventricle Size Covariate

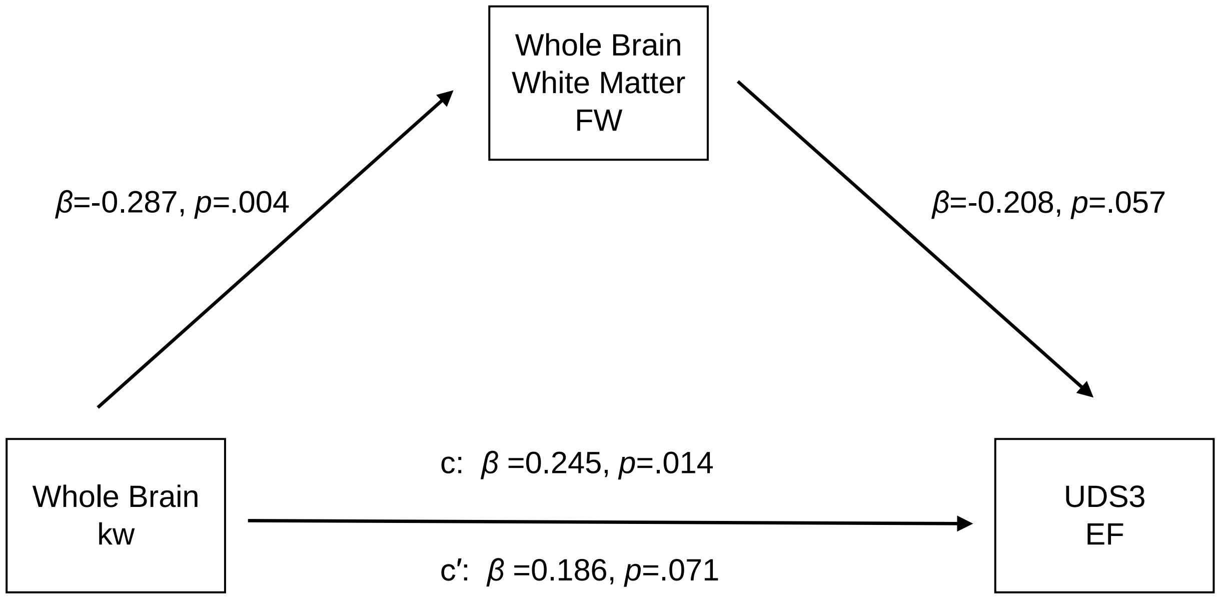

Note. The association between whole brain kw and executive function composite score, with whole brain white matter free water as a mediator. An indirect effect was observed for the models, but not a direct effect. Covariates included age, sex, and education.
